# Ferric pyrophosphate citrate: interactions with transferrin

**DOI:** 10.1007/s10534-018-0142-2

**Published:** 2018-10-11

**Authors:** Raymond Pratt, Garry J. Handelman, Thomas E. Edwards, Ajay Gupta

**Affiliations:** 1 Rockwell Medical, Wixom, MI USA; 2 University of Massachusetts Lowell, Lowell, MA USA; 3 Beryllium Discovery Corp, Washington, Bainbridge Island USA; 4 University of California Irvine, Orange, CA USA

**Keywords:** Iron, Transferrin, Crystal structure, Ferric pyrophosphate citrate, Kinetics

## Abstract

There are several options available for intravenous application of iron supplements, but they all have a similar structure:—an iron core surrounded by a carbohydrate coating. These nanoparticles require processing by the reticuloendothelial system to release iron, which is subsequently picked up by the iron-binding protein transferrin and distributed throughout the body, with most of the iron supplied to the bone marrow. This process risks exposing cells and tissues to free iron, which is potentially toxic due to its high redox activity. A new parenteral iron formation, ferric pyrophosphate citrate (FPC), has a novel structure that differs from conventional intravenous iron formulations, consisting of an iron atom complexed to one pyrophosphate and two citrate anions. In this study, we show that FPC can directly transfer iron to apo-transferrin. Kinetic analyses reveal that FPC donates iron to apo-transferrin with fast binding kinetics. In addition, the crystal structure of transferrin bound to FPC shows that FPC can donate iron to both iron-binding sites found within the transferrin structure. Examination of the iron-binding sites demonstrates that the iron atoms in both sites are fully encapsulated, forming bonds with amino acid side chains in the protein as well as pyrophosphate and carbonate anions. Taken together, these data demonstrate that, unlike intravenous iron formulations, FPC can directly and rapidly donate iron to transferrin in a manner that does not expose cells and tissues to the damaging effects of free, redox-active iron.

## Introduction

Iron supplements for intravenous (IV) application contain polymeric iron hydroxyl/oxy nanoparticles, often of mixed iron electrovalence, with a carbohydrate coating to control particle size and toxicity (Gupta 2016). These include the iron carbohydrate complexes iron dextran, iron sucrose, sodium ferric gluconate, and ferumoxytol; these complexes all contain an iron-oxyhydroxide/oxide central core with a carbohydrate shell (Girelli 2018). These nanoparticles require uptake by macrophages over a period of 4–17 h in the reticuloendothelial system to release the iron (Geisser and Burckhardt 2011; Koskenkorva-Frank 2013). During that delay period prior to uptake, these nanoparticles persist in the bloodstream. Once the complexes are degraded, iron is either stored within the macrophages or released into the plasma via the iron transporter ferroportin, where it binds to the iron transport protein, transferrin (Geisser and Burckhardt 2011). High iron levels in macrophages can induce a pro-inflammatory phenotype (Recalcati et al. 2012), which has been shown to impair wound healing (Sindrilaru et al. 2011), promote blood vessel wall calcification (Kamanna et al. 2012), and impair phagocytic function, potentially increasing the risk of infection (Ichii et al. 2012). In addition, iron-carbohydrate complexes contain a proportion of free iron that can be donated directly to transferrin until iron binding capacity is exceeded, resulting in non-transferrin bound iron (NTBI) (Gupta et al. 2016). NTBI is a potentially toxic form of iron, due to its potential to induce reactive oxygen species (ROS) and cellular damage (Brissot et al. 2012). In a prospective study in patients on hemodialysis receiving IV iron, currently approved IV iron products were associated with increased exposure to NTBI as well as increased pro-inflammatory cytokines and ROS (Pai et al. 2011).

Ferric pyrophosphate citrate (FPC; Triferic) is an iron(III)-citrate-pyrophosphate oligomeric complex that does not contain a carbohydrate shell and that efficiently delivers iron to plasma transferrin. Previously in this issue, we reported the physicochemical characteristics of FPC in the solid state and in solution. FPC consists of a unique iron(III)-citrate-pyrophosphate ternary complex oligomeric structure, stable for extended periods in aqueous solution. FPC was approved by the US Federal Drug Administration (FDA) in 2015 as the first carbohydrate-free, water-soluble, complex iron supplement suitable for rapid administration via dialysate in adult patients requiring hemodialysis (HD) for chronic kidney disease (CKD) (Rockwell Medical Inc 2018). In two multicenter, randomized, placebo-controlled, phase 3 clinical trials FPC was shown to safely replace iron and maintain hemoglobin levels, without increasing iron stores, in patients undergoing chronic HD (Fishbane et al. 2015).

In a recent pharmacokinetic study, administration of FPC did not increase NTBI and did not increase markers of oxidative stress in healthy volunteers (Pratt et al. 2017). Similarly, in the phase 2 PRIME study, FPC maintained hemoglobin levels in patients on hemodialysis without inducing markers of inflammation and oxidative stress (Gupta et al. 2015). In the above studies, increase in serum iron during infusion of FPC is accompanied by concomitant parallel decline in serum unsaturated iron binding capacity (UIBC) in a dose-dependent manner, indicating that FPC donates iron directly to apo-transferrin (apo-Tf) in the bloodstream, bypassing the reticuloendothelial system. The goal of this study was to evaluate the mechanism by which FPC donates iron to human apo-Tf. We show that FPC can bind directly to Apo-Tf, which promotes rapid transfer of iron, thus limiting exposure of NTBI and its associated negative effects.

## Materials and methods

### In vitro binding of FPC to apo-transferrin

The uptake of iron by human apo-Tf was measured by the change of transferrin absorbance at 462 nm (molar extinction increase for saturated human transferrin = 4860) (Frieden and Aisen 1980). Measurements were done on a Shimadzu-UV-1601 recording spectrophotometer (Shimadzu Scientific, Columbia, MD).

Human apo-Tf (Sigma, St. Louis, MO, Product # T-1147), was dissolved in 0.05 M Tris buffer, pH 7.4, with 20 mM sodium bicarbonate. It contained < 1% iron content, as determined by iron assay with ferrozine reagent.

FPC (Rockwell, Lot #4004), and food-grade iron pyrophosphate (Dr. Paul Lohmann GmbH KG, Emmerthal, Germany) were dissolved in Tris/bicarbonate buffer. Ferric-nitrilotriacetate (Fe-NTA) was prepared from ferric nitrate (Fisher Scientific, Pittsburgh, PA) and nitrilotriacetic acid (Sigma), following the protocol of Bates et al. (Bates et al. 1967).

The apo-Tf solution (1 ml) was equilibrated at 37 °C in a quartz cuvette, the iron-complex solution (20 μL) was added, and kinetic measurements started within 10 s of mixing. Data were recorded digitally at 1–s intervals, and the reaction was monitored until steady-state measurements were achieved.

### Purification of transferrin for crystallography

Human apo-Tf samples were obtained from commercial sources: human apo-Tf (pro-325) from ProSpec and human apo-Tf (T2036) from Sigma Aldrich. Each human apo-Tf sample was purified using a Q-Sepharose FF ion exchange column in 25 mM HEPES buffer, pH 8.0. The protein was eluted using a linear salt gradient (0–200 mM NaCl) and buffer exchanged into 10 mM HEPES buffer pH 7.4. The final purity of both transferrin preparations was greater than 95%, as determined by capillary gel electrophoresis.

### Crystallization of human apo-transferrin and FPC-bound transferrin

Sitting drop vapor diffusion crystallization experiments were performed on human transferrin at 56 mg/mL (0.76 mM) in the absence and presence of 2 mM FPC using Compact Jr crystallization plates from Rigaku Reagents at 16 °C with 0.4 µL protein and 0.4 µL precipitant equilibrated against 80 µL reservoir. Crystals were obtained from several conditions. Apo-Tf crystals were only obtained from the ProSpec protein and not the Sigma Aldrich protein. Crystals of human Apo-Tf (ProSpec) were obtained from the JCSG + screen condition G2 (0.02 M MgCl_2_, 0.1 M HEPES pH 7.5, 22% v/v polyacrylic acid 5100 sodium salt, supplemented with 15% v/v ethylene glycol as cryo-protectant). Crystals of human transferrin in complex with FPC were obtained from the Morpheus screen (Gorrec 2009) condition d4 (12.5% PEG 1000, 12.5% PEG 3350, 12.5% MPD, 0.02 M each 1,6-hexanediol, 1-butanol, (RS)-1,2-propanediol, 2-propanol, 1,4-butanediol, 1,3-propanediol, 0.1 M MES/imidazole pH 6.5) for the ProSpec sample and the Morpheus screen condition F5 (10% PEG 20,000 20% PEG MME 550, 0.02 M each d-glucose, d-mannose, d-galactose, l-fucose, d-xylose, *N*-acetyl-d-glucosamine, 0.1 M MOPS/HEPES-Na pH 7.5) for the Sigma Aldrich sample. The apo-Tf crystals were visually clear, whereas the FPC-containing Tf samples were visually red.

### Structure determination of human apo and FPC-bound transferrin

X-ray diffraction data sets were collected at the Advanced Photon Source. Data were reduced with XDS/XSCALE (Kabsch 2010). The crystal structure of human apo-Tf was solved by molecular replacement in Phaser (McCoy et al. 2007) using the human apo-Tf structure(Wally et al. 2006), and the crystal structure of human FPC-bound transferrin was solved using the human bismuth-bound transferrin structure with the metals, carbonate, and NTA removed (Yang et al. 2012). The structure was obtained after numerous rounds of refinement in REFMAC5 (Murshudov et al. 2011) and manual model building in Coot (Emsley and Cowtan 2004) with a final round of refinement in Phenix (Adams et al. 2002). The structure was assessed for geometry and validated in MolProbity (Chen et al. 2010). The crystal structure of human transferrin bound to FPC has been deposited into the Protein Data Bank with accession code PDB ID 6CTC.

## Results

### Kinetics of Fe delivery to serum transferrin

**Fig. 1:**
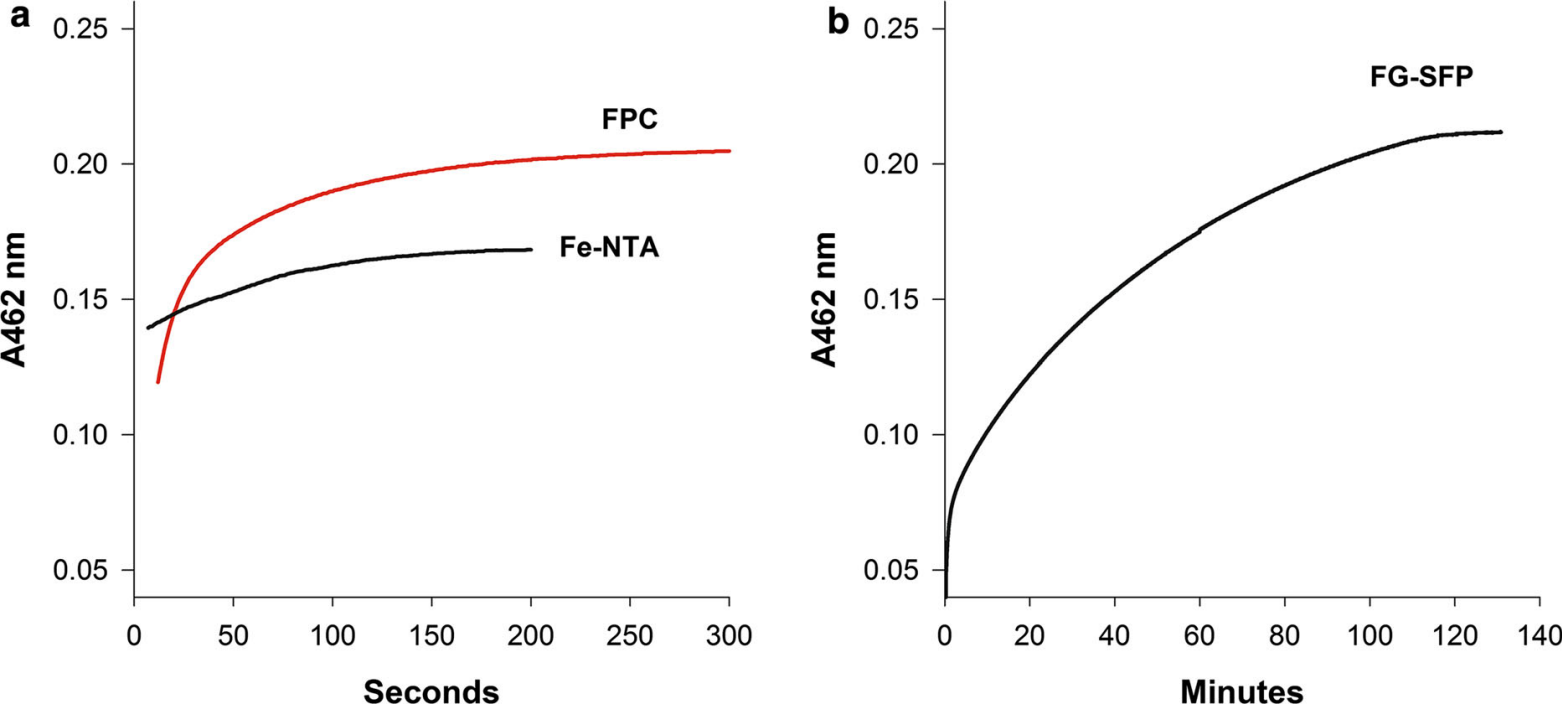
Kinetics of FPC and SFP binding to apo-transferrin. The absorbance at 462 nm was monitored to measure ferric iron uptake to human apo-transferrin from FPC, Fe-NTA, as a positive control (**a**), and food grade SFP (**b**)

Figure 1a shows an overlay of the UV absorbance spectrum for FPC binding to human apo-transferrin and for the binding of the Fe-NTA control. As expected, the Fe-NTA control showed a rapid increase in absorbance at 462 nm. FPC achieved 100% iron loading of human apo-transferrin within 10 min. Maximum absorbance values using iron-phosphate complexes ranged from 0.2 to 0.25, and were comparable to the theoretical maximum, with a 2–1 stoichiometry of iron to apo-transferrin. Rates of iron uptake were similar between Fe-NTA and FPC, suggesting that FPC can donate iron directly to human apo-Tf with fast-binding kinetics. Food-grade SFP donated iron much slower with time to maximal loading of 1.5 h (Fig. 1b).

### Crystal structure of human apo-transferrin

Crystals of human apo-Tf obtained from the ProSpec sample were purified by ion exchange chromatography and resulted in a *P*-centered orthorhombic crystal form with unit cell dimensions similar to a previous report (Wally et al. 2006). An initial data set and structure were obtained at 3.6 Å-resolution (Table 1). Both molecules in the asymmetric unit exhibited an open conformation in both the N-lobe and C-lobe, consistent with the previously solved apo-transferrin structure (Wally et al. 2006). Examination of the electron density in the active sites of all four lobes did not reveal any significant difference density peaks, indicating that the purified protein reagent is indeed apo and does not contain iron.

**Table 1:** Crystallographic statistics for human transferrin data sets

	Apo	FPC
Beamline	APS 21 ID-G	APS 21 ID-F
Collection date	26-June-2013	3-July-2013
Space group	*P*2_1_2_1_2_1_	*P*2_1_2_1_2_1_
Unit cell	*a *= 85.08 Å, *b *= 103.06 Å, *c *= 200.73 Å, α = β = γ = 90°	*a *= 74.37 Å, *b *= 90.16 Å, *c *= 110.43 Å, α = β = γ = 90°
Solvent content	58.1%	50.2%
V_m_	2.94 Å^3^/Da	2.47 Å^3^/Da
Resolution	50–3.6 Å (3.85–3.60 Å)	50–2.6 Å (2.65–2.60 Å)
I/σ	7.0 (3.6)	22.2 (4.2)
Completeness	93.9% (93.6%)	99.9% (100%)
R_merge_	0.148 (0.381)	0.045 (0.484)
Multiplicity	3.5 (3.5)	7.0 (7.2)
Unique reflections	19,841 (1,442)	23,450 (1713)
Mosaicity	0.3–0.9	0.5
Refinement		
R		0.1801
R_free_		0.2532
Validation		
Ramachandran favored		94.0%
Ramachandran outliers		0.15%
Rotamer outliers		0.19%
Clash score		7.96
Molprobity score		1.83

### Crystal structure of human transferrin bound to FPC

**Fig. 2:**
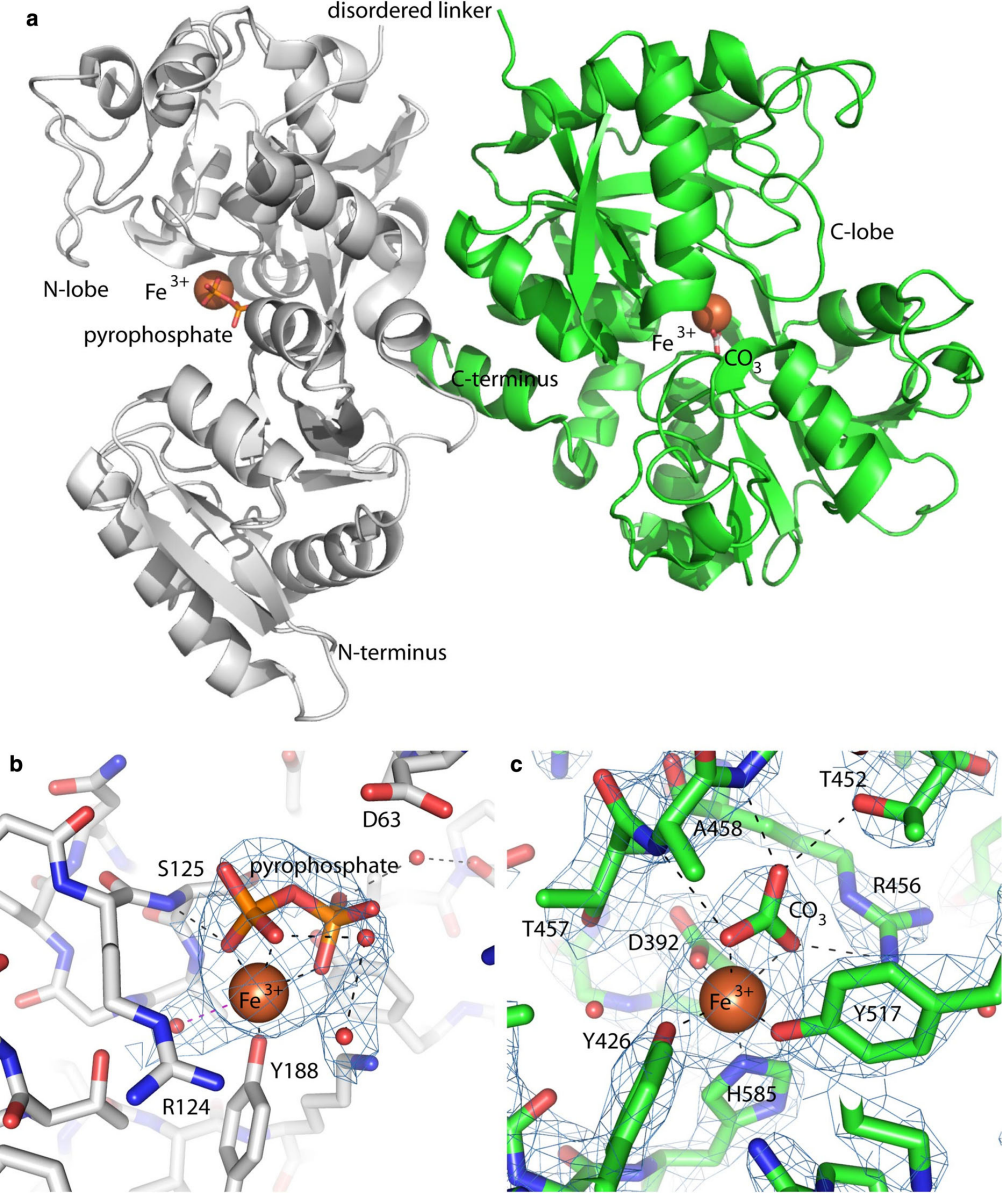
Crystal structure of FPC-bound transferrin. **a** 2.6-Å resolution co-crystal structure of human transferrin bound to FPC shown with the N-terminal lobe in gray ribbons and the C-terminal lobe in green ribbons. Iron atoms are shown as orange spheres. Pyrophosphate and carbonate are shown in sticks rendering. The linker between the N-lobe and the C-lobe was disordered and is labeled, and the N- and C-termini are also labeled. **b** The N-lobe bound to iron and pyrophosphate. **c** The C-lobe bound to iron and carbonate. 2|F_0_| − |F_c_| electron density maps are shown in blue mesh contoured at 1.0 σ and individual amino acids are labeled

Crystals of human transferrin bound to FPC were obtained and resulted in 2.6-Å resolution data sets for both the Sigma-Aldrich and ProSpec samples. Because the crystal structure of the ProSpec apo-transferrin demonstrated that the sample was truly apo-Tf, we proceeded with structure determination of the ProSpec human transferrin protein bound to FPC (Table 1). The Sigma-Aldrich sample-obtained transferrin/FPC structure was otherwise identical, and the analysis below is also identical. Both the N- and C-lobe iron-binding sites contain an iron atom, indicating that FPC can donate iron to both lobes of human apo-transferrin. Globally, the structure of FPC-bound transferrin, shown in Fig. 2a, more closely resembles that of bismuth-NTA- and iron-sulfate-bound transferrin (Yang et al. 2012), in which the N-lobe is partially open and the C-lobe is fully closed, than that of the fully open, apo-Tf form(20) or the fully closed diferric-Tf form (Noinaj et al. 2012).

The electron density map at the iron binding site in the N-lobe contained a strong difference density peak, indicative of an iron atom. The difference density could not be accounted for by iron alone or a water-coordinated iron; however, we were able to model an iron atom with a pyrophosphate molecule with good confidence. In addition to making three coordinate bonds to the iron atom, the pyrophosphate molecule forms direct hydrogen bonds with the backbone amide of S125 and the side chain of Y95 as well as water-mediated hydrogen bonds with the backbone amide of A64 and the carbonyl of P247 (Fig. 2b). The position of pyrophosphate effectively blocks D63 and H249 in the N-lobe from approaching the iron atom to create a fully encapsulated iron as observed in a previously reported diferric structure. (Noinaj et al. 2012) The C-lobe contains a well encapsulated iron atom coordinated by D392, Y426, Y517, H585, and carbonate, like other iron-bound C-lobe structures described in the literature (Fig. 2c). (Noinaj et al. 2012; Yang et al. 2012) The C-lobe iron density is the strongest electron density feature (10 σ in the 2|F_0_| − |F_c_| map), whereas the N-lobe iron density is one of the strongest electron density features (3.5 σ in the 2|F_0_| − |F_c_| map), consistent with previous reports that the C-lobe has a higher affinity for iron than the N-lobe. (Cannon and Chasteen 1975; Princiotto and Zapolski 1975).

## Discussion

Iron is critical to several biological processes due to its inherent redox properties; however, these properties also make iron potentially toxic. If not well controlled, iron can generate ROS and damage cells and tissues. Therefore, the body has developed mechanisms to regulate iron mobilization and limit exposure to free iron. The primary means by which iron is transported in the circulation is bound to the protein transferrin, which contains two iron-binding sites that bind tightly, but not covalently, to iron (III) and deliver it to cells and tissues throughout the body. (Aisen et al. 1978; Luck and Mason 2012) Labile plasma iron (LPI) is an indicator of redox-active iron and is believed to be a toxic species. Iron bound to transferrin does not contribute to LPI, and LPI is not present in the bloodstream until transferrin iron binding capacity is exceeded. (Esposito et al. 2003; Pratt et al. 2017).

In patients with or at risk for iron-deficiency anemia, iron supplementation may be necessary to replace iron losses and maintain iron levels. While oral iron supplements are available, these agents may be poorly absorbed in the intestine or result in gastrointestinal side effects, and therefore may not be suitable for some patients, including most patients undergoing hemodialysis or receiving parenteral nutrition. (Fudin et al. 1998; Macdougall et al. 1996; Markowitz et al. 1997) For such patients, IV iron supplementation has become standard of care. While there are several IV iron products available, they all have a similar structure—an iron core surrounded by a carbohydrate moiety. (Girelli et al. 2018) These iron/carbohydrate complexes must first be processed by the reticuloendothelial system before the iron can be loaded onto transferrin and used throughout the body. (Koskenkorva-Frank et al. 2013) Following intravenous administration, the complexes are endocytosed by macrophages, where they are degraded, and iron is either released into the plasma or stored. In addition, the complexes can break down in the bloodstream, releasing free NTBI into circulation. (Pai et al. 2011).

Excess iron that is not released into the plasma is stored in macrophages in the form of ferritin. (Koskenkorva-Frank et al. 2013) IV iron can have profound effects on macrophage phenotype and function, leading to inflammation, endothelial cell dysfunction, and increased risk of infection. (Ichii et al. 2012; Kamanna et al. 2012; Recalcati et al. 2012; Sindrilaru et al. 2011) In animal studies, a stepwise accumulation of iron in first the spleen and then the liver has been demonstrated, with an associated decrease in dietary iron absorption and increase in the pro-inflammatory hormone hepcidin. (Theurl et al. 2005) Hepatic imaging and biopsy studies of hemodialysis patients have revealed that most patients have higher than normal iron stores and that hepatic iron stores correlate with IV iron dose. (Rostoker et al. 2016) Patients with concomitant inflammatory states such as dialysis patients have elevated levels of serum hepcidin, which promotes iron sequestration, and thereby interferes with utilization of iron provided during by the iron-carbohydrate formulations. IV iron administration leads to marked increase in serum hepcidin, further promoting macrophage iron sequestration. Therefore, in states of inflammation there is an unmet need for a parenteral iron product that can donate iron directly to Tf, bypassing the reticuloendothelial system. Previous attempts at parenteral administration of soluble iron salts that could donate iron directly to Tf were abandoned due to serious toxic reactions attributed to massive and rapid release of NTBI. Therefore, a novel parenteral iron salt has been developed in which iron is tightly complexed to the anion ligand. (Gupta and Crumbliss 2000) FPC is the first iron salt suitable for parenteral administration. FPC does not contain a carbohydrate moiety and has high solubility and stability, in part due to its distinct structures in solid and solution phases, which have been described previously in this issue. In short, FPC consist of an iron (III) atom coordinated by citrate and pyrophosphate anions. FPC belongs to a class of iron salts known as soluble ferric pyrophosphates (SFP). The difference between FPC and food grade SFP is related to the coordination of iron to citrate and pyrophosphate which results in high aqueous solubility. FPC is manufactured to pharmaceutical specifications while SFP is less well characterized. Kinetic analyses of iron (III) pyrophosphate have demonstrated that the complex can directly bind to and donate iron to apo-transferrin.(Cowart et al. 1986) In addition, several organic phosphates, including pyrophosphate, ATP, GTP, and 2,3-diphosphoglycerate have been shown to aid in the transfer of iron from transferrin to ferritin and for the transfer of iron across the mitochondrial membrane. (Konopka et al. 1981; Morgan 1977; Nilsen and Romslo 1984) In this study, we show via biochemical and structural analysis that FPC can directly donate iron to transferrin. Kinetic analysis of FPC/transferrin interaction reveals that FPC donates iron directly to transferrin with fast binding kinetics. The iron donation from food grade SFP is approximately 60-fold slower than FPC. This difference may be important when considering the delivery of iron via dialysate.

We also solved the crystal structure of FPC-bound transferrin. The structure of FPC-bound transferrin is reminiscent of that of bismuth-NTA- and iron-sulfate-bound transferrin, in which the N-lobe is partially open, and the C-lobe is fully closed. (Noinaj et al. 2012; Yang et al. 2012) The structure reveals that FPC donates iron to both the N- and C-lobe iron-binding sites within transferrin. Both iron atoms are fully encapsulated within the binding sites, and thus protected from generating labile plasma iron. In the N-lobe, iron is coordinated by several residues within transferrin as well as by pyrophosphate, which comes from FPC. The pyrophosphate anion is bound on the far side of the iron atom and may usher it onto transferrin. In the C-lobe, the iron atom is fully encapsulated by several protein residues and a carbonate anion.

Based on these results, we propose that FPC directly donates iron to transferrin in a manner that is not expected to lead to NTBI, the production of ROS, or the oxidative stress and inflammation that have been associated with IV iron formulations. (Geisser and Burckhardt 2011; Pai et al. 2011) Our data are consistent with previous clinical studies that show that FPC does not generate NTBI or increased hepcidin levels in healthy volunteers (Pratt et al. 2017) and does not increase markers of oxidative stress among hemodialysis patients. (Gupta et al. 2015) In the phase 2 PRIME trial, there was no statistically significant differences in biomarkers for oxidative stress (malondialdehyde) or inflammation (interleukin-6) in hemodialysis patients treated within the FPC vs placebo during a 9-month study period. (Gupta et al. 2015) Pharmacokinetics analysis of FPC showed that all the administered iron complexes with transferrin and that it is rapidly cleared from the plasma, with a mean apparent terminal-phase half-life of 1.2 h. (Pratt et al. 2017) Therefore, FPC is not expected to accumulate or lead to iron overload.

As a unique iron compound, FPC offers a promising new means for providing iron to patients with anemia caused by inflammation due to hepcidin-induced iron sequestration. Such patients include those dependent on dialysis or parenteral nutrition. Clinical studies have demonstrated that FPC reliably delivers iron while maintaining hemoglobin levels in patients with hemodialysis-dependent CKD with a safety profile that is similar to that of placebo (Fishbane et al. 2015). FPC has also been shown to reduce the erythropoiesis-stimulating agent and iron requirements needed to maintain hemoglobin concentration. (Gupta et al. 2015).
